# Detection of Mismatch Repair Deficiency in Endometrial Cancer: Assessment of IHC, Fragment Length Analysis, and Amplicon Sequencing Based MSI Testing

**DOI:** 10.3390/cancers16233970

**Published:** 2024-11-26

**Authors:** Peter Sowter, Richard Gallon, Christine Hayes, Rachel Phelps, Gillian Borthwick, Shaun Prior, Jenny Combe, Holly Buist, Rachel Pearlman, Heather Hampel, Paul Goodfellow, D. Gareth Evans, Emma J. Crosbie, Neil Ryan, John Burn, Mauro Santibanez-Koref, Michael S. Jackson

**Affiliations:** 1 Faculty of Medical Sciences, Newcastle University, Newcastle upon Tyne NE1 7RU, UK; ptrswtr188@gmail.com (P.S.); richard.gallon@newcastle.ac.uk (R.G.); christine.hayes@newcastle.ac.uk (C.H.); rachel.phelps@newcastle.ac.uk (R.P.); gillian.borthwick@newcastle.ac.uk (G.B.); jennifer.combe@hotmail.com (J.C.); john.burn@newcastle.ac.uk (J.B.); mauro.santibanez-koref@newcastle.ac.uk (M.S.-K.); 2 Newcastle Hospitals NHS Foundation Trust, Newcastle upon Tyne NE1 4LP, UK; h.buist@nhs.net; 3 Department of Internal Medicine, Wexner Medical Center, Ohio State University, Columbus, OH 43210, USA; rachel.pearlman@osumc.edu (R.P.); hhampel@coh.org (H.H.); 4 Department of Obstetrics and Gynecology, Wexner Medical Center, Ohio State University, Columbus, OH 43210, USA; paul.goodfellow@osumc.edu; 5 Division of Evolution Infection and Genomic Science, University of Manchester, Manchester M13 9PL, UK; gareth.evans@mft.nhs.uk; 6 Division of Gynaecology, Manchester University NHS Foundation Trust, Manchester M13 9WL, UK; emma.crosbie@manchester.ac.uk; 7 Division of Cancer Sciences, St Mary’s Hospital, University of Manchester, Manchester M13 9WL, UK; 8 College of Medicine and Veterinary Medicine, The University of Edinburgh, Edinburgh EH16 4SB, UK; neil.ryan@ed.ac.uk

**Keywords:** endometrial cancer, mismatch repair deficiency, immunohistochemistry, microsatellite instability, Lynch syndrome

## Abstract

**Background/Objectives:** Mismatch repair (MMR) deficiency can be indicative of Lynch syndrome (LS) and guide treatment with immune checkpoint inhibitors. Colorectal cancers (CRCs) and endometrial cancers (ECs) are routinely screened to identify LS, primarily using immunohistochemistry (IHC) or microsatellite instability (MSI) testing, but concordance between these methods is variable in ECs. Here, we investigate this variability in 361 ECs from the Ohio OCCPI/OPTEC (*n* = 196) and Manchester PETALS (*n* = 165) trials, where concordance between assays differed significantly. **Methods:** Samples were re-tested using the amplicon-sequencing-based Newcastle MSI assay (NCL_MSI), and analysed with respect to existing IHC, MSI and MLH1 promoter hypermethylation data. **Results:** NCL_MSI showed consistency with the Ohio results (94% and 97% concordance with IHC and original MSI assays, respectively) and increased concordance within the Manchester cohort from 78% to 86% (MSI) and 84% (IHC). Among discordant Manchester samples, NCL_MSI was significantly associated with *MLH1* promoter methylation status (*p* = 0.0028) and had the highest concordance with methylation, (62/69 samples, 90%), indicating utility as a screening tool in this tumour type. However, tumours with germline *MSH6* defects were only detected efficiently with IHC; seven out of eight LS tumours classified as MSS by either MSI assay had isolated MSH6 loss, compared to four out of twelve classified as MSI-H by both (*p* = 0.028). Furthermore, reduced MSI signal was observed in tumours with isolated MSH6 loss (*p* = 0.009 Ohio, *p* = 6.2 × 10^−5^ Manchester) and in both ECs and CRCs with germline defects, although this only reached significance in CRCs (*p* = 0.002). **Conclusions:** These results provide further evidence that ECs with MSH6 loss in particular and LS tumours in general have an attenuated MSI signal, providing support for current guidelines specifically recommending IHC for LS detection and immune checkpoint therapy assessment in EC.

## Simple Summary

Tumours of the colon and endometrium (lining of the womb) often have defects in genes that repair our DNA. Identifying these tumours is important for patient treatment and identifying Lynch syndrome (LS), an inherited cancer predisposition. However, the two methods most commonly used to identify these defects often give inconsistent results for endometrial tumours. Here, we investigate these inconsistencies by re-analysing 361 tumour samples from clinical trials with a third assay and establish that they are largely due to false positives in one assay (immunohistochemistry) and false negatives in the other (Promega MSI assay). In addition, the DNA repair defects seem to have a reduced impact within endometrial tumours. However, we find that tumours with defects in a gene called *MSH6*, especially when associated with LS, are only detected efficiently with immunohistochemistry. This supports current guidelines specifically recommending the use of this method for endometrial tumours.

## 1. Introduction

The detection of mismatch repair deficiency (MMRd) is of critical importance regarding the care of people with cancer and their families as it is a primary determinant of the efficacy of immune checkpoint blockade therapy [1,2] and helps to identify individuals with Lynch syndrome (LS) who, along with affected family members, can benefit from enhanced surveillance and chemoprevention [3,4].

LS, the most common inherited cancer syndrome, is characterised by a predisposition to a range of tumours, with colorectal cancer (CRC) and endometrial cancer (EC) being the most common [5,6]. It is caused by heterozygous pathogenic variants affecting one of four genes central to the mismatch repair (MMR) pathway: *MLH1*, *MSH2*, *MSH6,* and *PMS2.* Somatic loss of the second allele leads to DNA repair failure and the accumulation of potentially tumorigenic variants. Repetitive sequences are especially prone to error induced by replication slippage, resulting in the associated molecular phenotype of microsatellite instability (MSI) [7,8]. Tumour spectrum and penetrance varies extensively according to the gene affected [9], leading to the suggestion that LS be considered as four separate clinical entities [10].

Almost all CRCs and ECs are routinely screened in England to identify LS cases, primarily using immunohistochemistry (IHC) or MSI analysis [11]. The former involves analysis of the expression of all four MMR proteins in tumour sections [12]. The latter most commonly involves fragment length analysis of highly informative PCR-amplified microsatellite markers (FLA-PCR [13,14]) using, for example, the Promega MSI Analysis System V1.2, which involves visual assessment of variation at five mononucleotide repeat markers (Promega MSI, [15]). Melt curve analysis of MSI marker amplicons [16] and analysis of markers within high throughput/panel sequencing data [17,18] are also now used.

These methods vary in terms of cost, throughput, ease of use, and result interpretation [11,12,13]. For instance, IHC is readily integrated with hospital pathology services, can often identify the gene affected, and is robust to heterogeneity in tumour cell content, but it requires expert interpretation and can miss up to 6% of pathogenic variants that do not affect antibody binding [19]. As a result, there is currently no single gold standard method for MMRd detection short of whole-genome tumour sequencing, and tumour-specific guidelines for LS screening have been developed in the UK based on health economic evaluation [20,21], with IHC- or FLA-PCR-based MSI methods recommended for CRCs (https://www.nice.org.uk/guidance/dg27 [accessed on 17 November 2024]) and IHC specifically recommended for ECs (https://www.nice.org.uk/guidance/dg42 [accessed on 17 November 24]). Similarly, US and European medical professional organisations recommend the use of IHC over MSI to determine MMR status in EC patients being considered for immunotherapy [22,23].

One factor contributing to tumour-specific guidelines is that while IHC and MSI give highly concordant results in CRCs [24,25], test concordance in ECs is much more variable [11,26]. Some variability can be attributed to use of dinucleotide repeat markers in early MSI assays, which do not efficiently identify *MSH6* deficiency, common in EC [27,28,29]. Levels of MSI are also generally lower in EC relative to CRC [30,31], suggesting that current MSI assays lack sensitivity or need to be optimised for use in this tumour type [18].

We have recently developed an amplicon-sequencing-based MSI method [32,33], which has been adopted as the primary assay of MMRd in CRC in our NHS region—the Newcastle MSI assay (NCL_MSI). This uses a Bayesian classifier to dichotomise samples into microsatellite stable and unstable, based on length variation at 24 mononucleotide repeat loci. Here, we use this assay to analyse previously well-characterised clinical trial cohorts with distinct concordance levels between IHC and MSI [34,35,36] and assess the impact both of using a larger and more sensitive MSI marker panel [32] and using classification criteria specific for ECs. We also analyse the MSI signal within sporadic and LS tumours with respect to MMR protein loss and MMR gene affected.

## 2. Materials and Methods

### 2.1. Sample Details

A cohort of 200 EC DNAs were provided by the Ohio State University Comprehensive Cancer Center as two sets of 100 samples. Those in set 1 (training) were from individuals diagnosed with primary invasive EC between 2013 and 2016, prospectively enrolled onto the Ohio Colorectal Cancer Prevention Initiative (OCCPI [35]). Those in set 2 (validation) were from women for whom a hysterectomy or diagnostic biopsy had confirmed a newly diagnosed EC between 2017 and 2020 and who were enrolled on the Ohio Prevention and Treatment of Endometrial Cancer trial (OPTEC [34]). A second cohort comprised 191 resected tumour samples from women recruited to Manchester University NHS Foundation Trust gynaecological clinics between 2015 and 2017 following a diagnosis of EC, previously analysed in the Proportion of Endometrial Tumours Associated with Lynch Syndrome (PETALS) prospective study [37]. These were also divided into training (*n* = 95) and validation (*n* = 96) sets.

Both cohorts had similar numbers of mismatch repair proficient (MMRp) and deficient (MMRd) samples (~50:50) and included a small number of samples from LS patients (see Table S1). IHC results from all four MMR proteins were available for both cohorts. Results from the Promega MSI V1.2 assay (Promega Corporation, Madison, MI, USA) were available for all Manchester and OPTEC samples, and all OCCPI samples had clinical NGS MSI typing results, along with Promega MSI results from 76 samples (100% concordance between MSI methods). The MSI Low classification was treated as equivalent to MSS [38]. For validation sets, previous assay results and associated data were only provided after sequence analysis and initial NCL_MSI classification.

A further 35 CRCs derived from LS patients enrolled on the CaPP3 clinical trial (Trial number ISRCTN16261285) were obtained from the Cancer Prevention Programme bioresource, Newcastle University, and 56 unselected CRCs were obtained as anonymised discard FFPE clinical samples from the Newcastle NHS Genomics Laboratory Hub. All were derived from resected tumour blocks.

### 2.2. DNA Extraction, MSI Marker Panels, and MIP Amplification and Sequencing

Newcastle and Manchester cohort DNAs were extracted from 10-micron FFPE curls using the Gene Read DNA FFPE Kit (Qiagen, Hilden, Germany) and quantified using a QuBit 2.0 Fluorometer with QuBit dsDNA BR/HS Kits (Invitrogen, Paisley, UK). MSI marker panels (Table S2) and molecular inversion probe (MIP) sequences are described in [32,33,39] and in Table S3. Oligos were obtained from Metabion (Planegg, Germany). MIP preparation and amplification was performed according to the protocol outlined in [33] using 100 ng of template DNA. Amplicons were purified using AMPure XP beads (Beckman Coulter, Brea, CA, USA), diluted to 4 nM in 10 mM Tris pH 8.5, pooled in equal volumes, and sequenced as paired reads using MiSeq V3 kits (Illumina, SanDiego, CA, USA) to a depth of ~2000 reads per marker per sample using custom primers [40].

### 2.3. Sequence Analysis and MSI Classification

Sequencing reads were aligned against the hg19 human reference using BWA v0.7.17 [41] and classified using a naïve Bayes approach to produce an MSI score, as outlined in [39]. In brief, training cohorts were used to estimate the probabilities of observing a microsatellite deletion allele frequency (and allelic bias where a nearby SNP could be used to distinguish paternal and maternal alleles) for each MSI marker in MSS and MSI-H tumours, enabling the posterior probability of a new sample being MSS or MSI-H to be estimated. Assay scores are log_10_ odds that a sample is MSI-H versus MSS, with scores >0 classified as MSI-H and <0 as MSS. The MSI marker panels and scoring methods are collectively referred to here as the Newcastle_MSI assay (NCL_MSI).

### 2.4. Comparison of Tumour Cell Content with MSI Classification

Tumour cell content (TCC) estimates were available for OCCPI and Manchester samples. They were recorded in 10% increments for OCCPI but were not uniformly recorded for the Manchester samples, with specific as well as minimum, maximum, and range estimates used. For analysis, range estimates were arbitrarily recoded as the midpoint, and minimum/maximum values were recoded as 5% above/below the figure given. A single text entry “very sparse” was recoded to 4%, 1% below the lowest numeric value recorded.

### 2.5. Statistical Analyses

All analyses were performed in R v3.3.1. Graphs were generated using ggplot2 v3.5.1 within the tidyverse_2.0.0 package. Analyses of the distribution of variables between sample cohorts/groups were performed using Fisher’s exact tests. For analysis of the relative frequency of marker reference alleles (RAFs) between sample groups, frequencies were normalised with respect to medians in a reference group to allow comparison across different markers. A two-sided binomial test was used to assess whether there was a significant excess or reduction in the number of markers showing increased VAF with respect to the reference group. For convenience, we plotted the variant allele frequency (VAF = 1 − RAF). For groups containing small numbers of samples, such an approach may not reflect general characteristics of the group as it does not account for correlations between different microsatellites within a sample. We therefore used Beta regression (as implemented in the R package glmmTMB v1.1.9) and mixed models to investigate the significance of these changes, allowing for differences in instability across individuals and markers.

## 3. Results

The Ohio (OCCPI/OPTEC) and Manchester (PETALS) trials involved unselected series of EC patients, with MMRd tumour frequencies between 25% and 29% [34,35,36]. Concordance between IHC and MSI was uniformly high in the Ohio pathway [42,43] but was only 88% for the PETALS trial (*n* = 500), falling to 61% among MMRd samples [36]. The sample cohorts obtained for analysis with the NCL_MSI assay were enriched to contain approximately equal numbers of MMRd/MMRp samples.

We first amplified both cohorts using our CRC-trained molecular inversion probe (MIP) 24-marker MSI panel (NCL_MSI) [33]. Sequence data were successfully generated from 196/200 Ohio samples (received as purified DNAs), but 26/191 (14%) of the Manchester samples (received as paraffin embedded blocks) had to be excluded due to processing, amplification, or sequence quality issues (see Table S4). Analysis of the previously reported IHC and MSI statuses of these samples (Table 1) confirmed there was a difference in assay concordance between Ohio and Manchester cohorts (18%) that was highly significant (*p* = 1.2 × 10^−7^). MMR defects within the cohorts, in terms of genes affected and inheritance status, are summarised in Table S1.

### 3.1. NCL_MSI Is Highly Concordant with Ohio Assay Results

The concordance of the NCL_MSI results with the original assays and this method’s sensitivity and specificity relative to IHC (the method recommended for LS screening of ECs) are presented in Table 2, split by cohort (Ohio/Manchester) and dataset (training/validation). Results from both the Ohio training (OCCPI) and validation (OPTEC) datasets were consistent with the original studies, with concordance ranging from 94% to 98%. In the merged dataset (*n* = 196), NCL_MSI obtained 97% concordance with Ohio MSI results and 94% with IHC, similar to the concordance between the original assays (96%). NCL_MSI showed slight reductions in both sensitivity (93% vs 95%) and specificity (96% vs 97%) relative to IHC, but the differences were not significant.

### 3.2. NCL_MSI Increases Assay Concordance and Sensitivity in the Manchester Cohort

Concordance between the original assays was higher in the Manchester validation dataset than in the training dataset (82% vs 73%, Table 2), but the difference did not achieve statistical significance (*p* = 0.14). The NCL_MSI assay increased concordance with IHC in both datasets, reducing the difference between the two (85% vs 84%). In the merged dataset, concordance values with both original assays were similar (86% with MSI, 84% with IHC), concordance with IHC was improved (78% to 84%), and there was an increase in sensitivity of 11% (65% to 76%). However, these improvements were not significant (both *p* > 0.05).

### 3.3. Increasing Marker Number and Classifier Retraining Do Not Improve Assay Concordance

Although the NCL_MSI assay reduced the difference in concordance between IHC and MSI classifications in the Ohio and Manchester cohorts (Table 2), they remained significantly different between cohorts (original *p* = 5 × 10^−5^, NCL_MSI *p* = 0.002). Before analysing the Manchester data in more detail, we therefore tried to improve assay concordance further by increasing MSI marker number and by altering the training data used with the MSI classifier (Table S5).

We first scored both cohorts with an updated MIP panel of 56 highly discriminating MSI markers, known to improve the separation between classifier scores of MSS and MSI-H CRCs [32]. Data were successfully generated from all 196 Ohio samples and 156/165 Manchester samples. Reclassification resulted in small increases in concordance (94% to 96%) and sensitivity (93% to 96%) with respect to IHC in the Ohio cohort, and reductions in concordance (84% to 81%) and sensitivity (76% to 70%) in the Manchester cohort. None were statistically significant (*p* > 0.05). We also assessed the impact of training the 24-marker classifier using ECs, the appropriate tumour type. Again, only minor and non-significant changes were observed (Table S5). All subsequent analyses of the Manchester cohort were therefore performed using the original 24-marker NCL_MSI results.

### 3.4. Improved Concordance Mostly Affects IHC MMRd and Promega MSS Classifications

**Figure 1:**
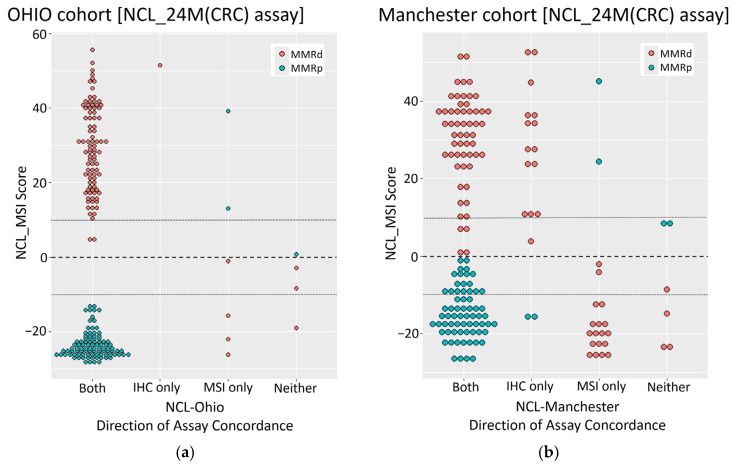
Concordance between NCL_MSI and original IHC/MSI results from both cohorts. (**a**) Ohio (*n* = 196). (**b**) Manchester (*n* = 165). X axis—concordance of NCL_MSI score obtained with the CRC-trained 24-marker panel. Y axis—NCL_MSI assay score (CRC-trained 24-marker panel). Sample colour indicates MMR status by IHC.

To investigate discordance between assays and cohorts in more detail, we plotted NCL_MSI scores with respect to the nature of assay concordance and MMR status as defined by IHC (Figure 1). In the Ohio cohort, the NCL_MSI was concordant with both original assays in 94% of cases, with MSI-H and MSS samples clearly dichotomised by assay score. Of the eleven samples showing discordance between NCL_MSI and one or both of the original assay results, one agreed with IHC only, six with MSI only, and four with neither (Figure 1a; see Ohio dataset in Table S6). Only six samples (3%) gave scores within ten points of the classification threshold (dotted lines Figure 1a and Table S6).

In contrast, the Manchester samples were not so clearly dichotomised, with 28 (17%) giving NCL_MSI scores of between +10 and −10 (dotted lines in Figure 1b and Table S7). In total, 43 (26%) showed discordance between the NCL_MSI classification and one or both of the original assay results (Figure 1b; see Manchester dataset in Table S7). The majority of these (37) were classified as MMRd by IHC. Only six were discordant with both original assays (two classified as MSI-H, four as MSS by NCL_MSI). Most samples where the NCL_MSI assay was concordant with IHC only were classified as MSI-H by NCL_MSI but as MSS by Promega MSI (15/17, scores > 0). In contrast, most samples where the NCL_MSI assay was concordant with Promega MSI only were classified as MSS by both MSI analyses but as MMRd by IHC (18/20, scores < 0). This association between NCL_MSI status and direction of assay concordance was significant (*p* < 1 × 10^−5^), indicating a systematic difference in assay performance within this cohort.

### 3.5. Manchester Discordance Suggests Promega MSI False Negatives and IHC False Positives

**Figure 2:**
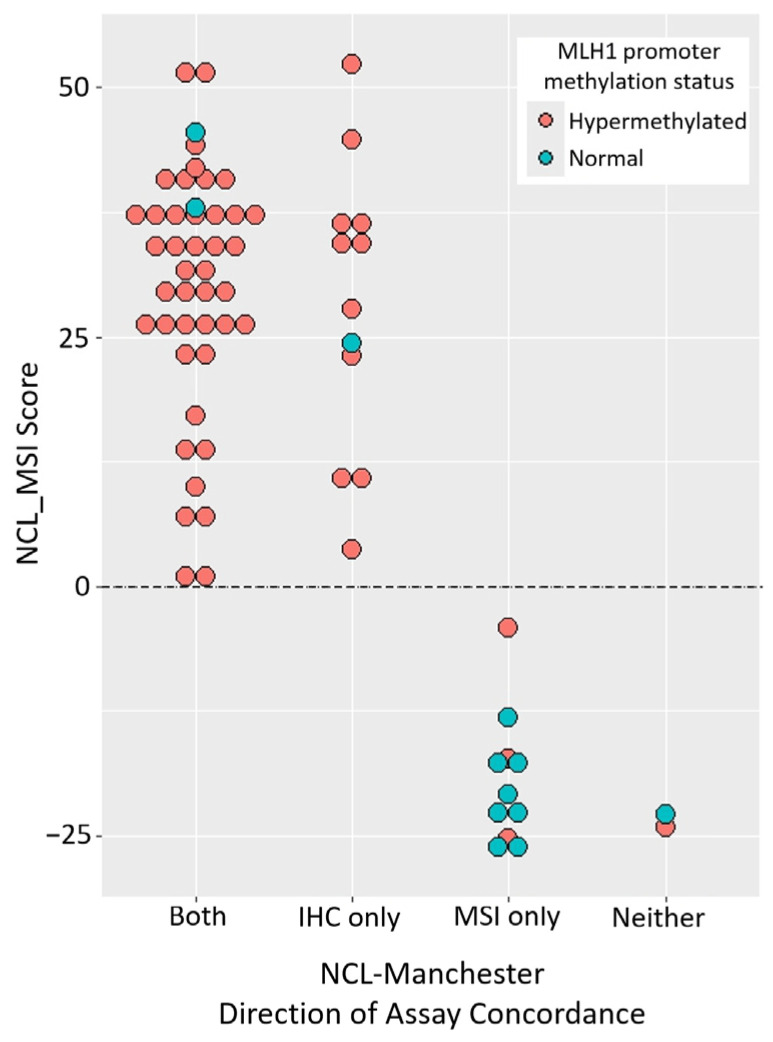
Promoter methylation and assay concordance within sporadic tumours showing loss of MLH1 protein expression by IHC (*n* = 69).

To try to resolve the origin of assay discordance in the Manchester cohort, we used *MLH1* promoter hypermethylation status as an independent measure of MMR status (truth set). This information was available for 92 samples, including 69 sporadic cases with loss of MLH1 protein staining (collated with respect to assay concordance in Table S8). Among sporadic samples with loss of MLH1 protein expression and discordant assay results (Figure 2), there was a significant association between NCL_MSI classification and *MLH1* promoter hypermethylation status (*p* = 0.0028). Specifically, 11/12 samples where NCL_MSI was concordant with IHC but not Promega MSI were both classified as MSI-H by NCL_MSI and hypermethylated, indicating probable Promega MSI false negatives. In contrast, 8/11 samples concordant with Promega MSI but not IHC were classified as MSS by both MSI assays and had normal methylation, indicating probable IHC false positives. This suggests that a minimum of 19 out of 69 sporadic samples with MLH1 loss (28%) may have been incorrectly classified by one or other assay in the original analysis. Furthermore, among these samples, NCL_MSI classification had the highest overall concordance with methylation status: 62/69 samples (90%), compared to 57/69 (83%) for IHC and 52/69 (75%) for Promega MSI (Figure 2).

### 3.6. Sample Tumour Cell Content Differs Between Cohorts and Impacts MSI Classification

As accuracy of MSI classification is known to be affected by tumour cell content (TCC), we reviewed the results in relation to estimates of TCC where available. This information was not recorded in the same way across the two cohorts (see methods), but a clear difference was apparent. Within the Ohio cohort, 85/87 (98%) of samples for which data were available had TCC estimates of 50% or above (Table S6), compared to only 47/96 (49%) within the Manchester cohort (Table S7). Furthermore, the lowest estimate was 40% in the Ohio samples, whereas 23 Manchester samples had a TCC of 20% or less, necessitating enrichment through macrodissection prior to DNA extraction, as per the PETALS protocol [36]. Manchester samples classified as MSS by Promega MSI were found to have a lower TCC than samples classified as MSI-H (*p* = 0.037, Mann–Whitney U test), but no difference was observed with respect to NCL_MSI classification (*p* = 0.11), although the trend was the same. This suggests that the lower TCC of the Manchester samples could be contributing to Promega MSI false negatives, despite enrichment. Furthermore, 12 samples were recorded as having patchy IHC staining within the Manchester cohort (Table S7), suggestive of subclonal MMRd, and these were found to be overrepresented in samples with MSI/IHC discordance (7/42 vs 5/123, *p* = 0.013).

### 3.7. Germline Cases Are Not Identified Efficiently by Either MSI Assay in Manchester Cohort

As LS detection is a major rationale for MMRd/MSI analysis, we next examined assay performance in tumours with germline pathogenic MMR mutations as a separate group (Table 3). In the Ohio cohort, all three assays identified five out of the six LS tumours as MMRd/MSI-H, with one (ECT192) identified only by IHC and one (ECT102) identified only by the two MSI assays. In the Manchester cohort, IHC identified all 14 LS tumours with known pathogenic germline variants, while 8/14 and 7/14 were classified as MSI-H by NCL_MSI and Promega MSI, respectively. There was disagreement between the MSI assays in three cases, and both classified five out of the eight tumours with isolated MSH6 loss as MSS.

Across tumours with pathogenic germline mutations there was a significant association between MSH6 loss and MSS classification (*p* = 0.028); isolated MSH6 loss was found in seven out of the eight tumours classified as MSS by either MSI assay, compared to four out of the twelve classified as MSI-H by both (Table 3). This suggested that LS-derived ECs, particularly those with MSH6 loss, are difficult to identify with MSI analyses and led us to investigate the association between somatic variation in microsatellite length and pathogenic germline MMR variants.

### 3.8. Variant Allele Frequencies Are Lower in Tumours from MMR Germline Defect Carriers

**Figure 3:**
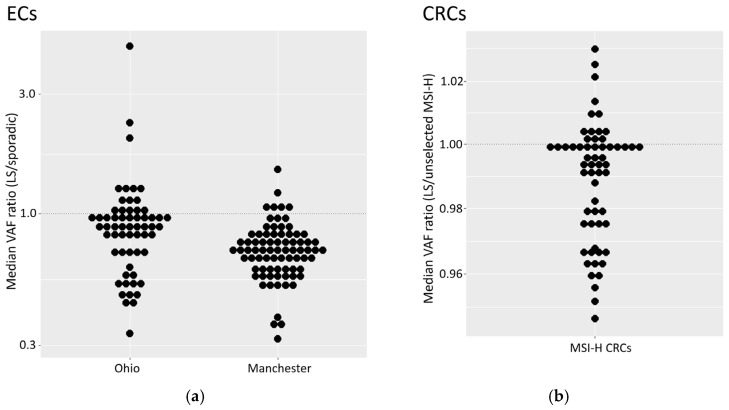
Magnitude of MSI signal in tumours with germline MMR defects. (**a**) Median VAFs for each marker in ECs with germline defects. Frequencies are normalised to the median for sporadic ECs for both the Manchester and Ohio cohorts. Markers from both the 24- and 56-marker panels were analysed. (**b**) Median VAFs for each marker in CRCs with germline defects. Median VAFs from an independent MSI marker panel (see methods) are shown for a cohort of 35 LS CRCs (see methods). Frequencies are normalised relative to median VAFs from 56 unselected CRCs.

To explore the relationship between tumour origin (germline/sporadic) and MSI levels, the median VAF for markers in tumours from germline carriers were normalised against the median VAF for each marker in sporadic tumours (Figure 3a; see methods). This mitigated inter-marker differences and allowed comparison across all MSI markers. A ratio below one represents a reduction in median VAF among germline tumours compared to sporadic ones.

Across both EC cohorts, MSI markers in samples with germline mutations showed lower variant allele frequencies than in sporadic samples (Figure 3a, *p* values: 5.8 × 10^−5^ for Ohio, 1.8 × 10^−15^ for Manchester). However, these results are expected to have been influenced by correlations between VAFs within samples (for example, due to variation in TCC). Analysis of each marker separately did not lead to statistically significant differences, possible due to the small number of germline cases in both cohorts (six and fourteen). We therefore explored whether similar results could be observed in a cohort of 35 MSI-H CRCs from LS patients previously analysed with 60 MSI markers (Tables S2 and S3), normalised to a cohort of 56 unselected MSI-H tumours (see methods). Beta regression, as well as a mixed model to account for sample specific effects within samples, identified a significant effect of LS status (Figure 3b, *p* = 0.002). This is consistent with the observations in EC, although the magnitude of the difference relative to the control group was greatly reduced. The unselected reference group was, however, treated as if they included no germline defects, an assumption that could reduce the apparent difference between groups.

### 3.9. Variant Allele Frequency Is Reduced in ECs with Isolated Loss of MSH6 Expression

**Figure 4:**
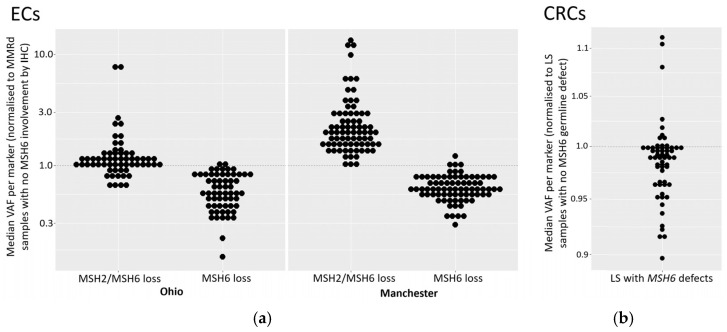
Magnitude of MSI signal in tumours with isolated MSH6 loss. (**a**) ECs with isolated loss of MSH6 expression and loss of both MSH2 and MSH6. Median VAF for each marker in ECs with loss of MSH6 only (*n* = 11 and 16) or loss of MSH6 and MSH2 (*n* = 13 and 7) is shown normalised to the median for MMRd samples identified as having no MSH6 involvement by IHC (*n* = 74 and 85). Markers from both the 24- and 56-marker panels were analysed. Samples with MSH6 loss have lower VAFs (*p* = 1.7 × 10^−12^ Man, 6.1 × 10^−15^ Ohio), while samples with MSH2 and MSH6 loss have higher VAFs (*p* = 2.1 × 10^−16^ Man, 3.8 × 10^−3^ Ohio). (**b**). CRCs from LS patients with known *MSH6* defects. Median VAFs from an independent MSI marker panel (see methods) in LS-derived CRCs with *MSH6* defects are shown normalised to the median for LS samples with defects in other MMR genes.

To investigate the relationship between variant allele frequencies (VAFs) and loss of MSH6 protein expression, we analysed median MSI marker VAFs, both in samples with isolated MSH6 loss and in samples lacking expression of MSH6 and MSH2, normalised to MMRd samples with no MSH6 loss (Figure 4a). Across both cohorts, ECs with isolated MSH6 loss had lower marker VAFs (*p* values: 1.0 × 10^−18^ for Manchester; 8.5 × 10^−16^ for Ohio), indicative of lower MSI. In contrast, in ECs with loss of both MSH2 and MSH6 expression, VAFs were higher (*p*-values: 1.8 × 10^−4^ and 1.0 × 10^−21^ for the Ohio and Manchester cohorts, respectively). A mixed effect model, to account for within-sample correlations, showed significant differences between samples with isolated MSH6 loss compared to samples without MSH6 loss in both cohorts (*p*-values: 0.009 and 6.2 × 10^−5^ for Ohio and Manchester, respectively). However, the differences did not reach statistical significance when comparing samples with MSH2 and MSH6 loss to samples without MSH6 loss (*p*-values 0.30 and 0.07 and for Ohio and Manchester, respectively).

We also sought to replicate this finding in the cohort of CRCs from LS patients (*n* = 35) by analysing median marker VAFs from tumours with MSH6 lesions, normalised to tumours with germline defects in other MMR genes. The results (Figure 4b) showed an excess of markers with median normalised VAFs below one in samples with *MSH6* germline lesions. While the small number of patients with *MSH6* defects in the CRCs (*n* = 3) precluded more sophisticated analyses, this result is consistent with the observations in ECs.

## 4. Discussion

We assessed the performance of the amplicon-sequencing-based NCL_MSI assay for use in ECs by re-analysing well-characterised tumour cohorts from prospective clinical trials and investigated the basis of previously reported discordance between IHC and MSI results. The NCL_MSI results were very consistent with the Ohio studies; concordance between NCL_MSI, IHC, and the original MSI results was >94%, comparable to the concordance between IHC and MSI in CRCs [12,19,24,43]. In the Manchester cohort, the NCL_MSI assay improved MSI sensitivity with respect to IHC by over 10% compared to the Promega MSI assay. *MLH1* promoter methylation data enabled two main groups of discordant samples to be identified (Figure 2): one suggesting Promega MSI false negatives (eleven with MLH1 loss and hypermethylation, identified as MSI-H by NCL_MSI but MSS by Promega MSI), the other IHC false positives (eight with MLH1 loss and normal methylation, identified as MSS by both MSI assays). This suggests that in the original Manchester analysis, up to 16% of tumours with MLH1 protein loss were Promega MSI false negatives, and up to 12% with loss of MLH1 were IHC false positives.

These results indicate that the NCL_MSI assay can be more sensitive than Promega MSI in some EC cohorts. One potential confounding factor is that the Ohio samples analysed were the same DNA aliquots used in the trials, whereas the Manchester samples were DNAs extracted from independent tissue curls taken from the original FFPE blocks analysed. This resulted in sample drop out and may have contributed to the higher discordance of the NCL_MSI assay with these samples. However, it cannot account for the significant skew with respect to IHC/Promega MSI classification or the significant association of NCL_MSI-H calls with *MLH1* promoter methylation status among discordant samples.

The results do not establish why assay concordance was significantly different between the original Ohio and Manchester analyses. All three trials (OCCPI, OPTEC, PETALS) had very similar enrolment and study protocols, but there are two potentially important differences.

First, although IHC methodology was comparable between studies, how tumours were dichotomised as MMRd or MMRp differed. Ohio samples were classified using College of American Pathologist (CAP) guidelines [44], where samples with equivocal or weak IHC staining (cut off defined as >1% positive nuclei) are classified as mismatch repair proficient (MMRp). In the Manchester cohort, classification of such samples was based on consensus guidelines [37], staining was repeated if results were inconclusive, and final classification was agreed by a team of expert pathologists. As a result, some samples with patchy IHC staining were classified as MMRd, others as MMRp (Table S7), with all being referred for germline MMR gene testing [36].

The potential impact of this difference can be understood by considering how IHC was conceptually embedded within the care pathways. In Ohio, it was complementary to MSI testing, with the expectation that any MMRd tumours missed as a result of the stringent IHC classification would be picked up by MSI testing [43]. In Manchester, IHC was deployed as a standalone assay to identify all potential LS cases, with anomalous results leading to referral for germline testing irrespective of MSI status [37]. In such a pipeline, a small number of IHC MMRd false positives can be tolerated and even considered desirable, as they are of minor consequence relative to a missed LS diagnosis and would be excluded in subsequent steps of the LS diagnostic pathway.

In this context, it is noteworthy that regions of tumours with patchy or subclonal MMR protein loss are known to have elevated frequencies of MSI [45] or somatic MMR gene mutations [46,47], and subclonal or weak MMR protein staining has recently been shown to account for much of the discordance between IHC and MSI assays [48,49]. This has led to the revision of US CAP recommendations to ensure that anomalous staining patterns are reported and interpreted consistently [48].

Second, a review of pathology reports established that some Manchester samples had low TCC, with a quarter having 20% or below and requiring enrichment prior to MSI analysis. Several were 5% or lower. Given that the limit of detection of Promega MSI is 10% tumour cells for CRC material [15], compared to ~3% for NCL_MSI [33], the significant association between low TCC and Promega MSS classification in the Manchester data strongly suggests that this contributed to Promega false negatives and highlights the importance of selecting material with high TCC for molecular testing in ECs.

Despite the increased sensitivity of the NCL_MSI assay in the Manchester cohort, it could not reliably identify tumours with inherited pathogenic MMR mutations, particularly cases with isolated MSH6 loss by IHC, classifying six out of eleven as MSS (Table 3). While low TCC could be a contributing factor in specific samples (PET31 in particular), three of the Manchester LS MSH6 samples classified as MSS by NCL_MSI had TCCs of 50% or above (Table S7). These results therefore support current guidelines recommending IHC as the primary assay for LS detection and assessment for immunotherapy treatment in EC patients (https://www.nice.org.uk/guidance/dg42, accessed on 17 November 2024 [22,23]).

However, IHC interpretation is subjective, can give equivocal results requiring repeat analysis, and relies on the expertise of the pathologist. Furthermore, MSI analysis can identify MMR mutations that do not affect antibody binding [50]. The assays are therefore complimentary, and use of both is desirable [43], potentially being of particular importance in ECs given the variation in concordance observed here and elsewhere (reviewed in [26]). We have recently developed a direct-to-sequencing multiplex PCR format of the NCL_MSI assay [51] to facilitate routine and high-throughput use. In addition, a further Promega MSI assay using longer microsatellite markers has also been developed (LMR MSI) that shows increased sensitivity for EC MMRd detection compared to the V1.2 assay [52], while integration of first-line gene/genome sequencing with these functional tests [53], or their adoption as alternatives [54], are strategies worthy of consideration.

The reduction in MSI signal we observed in ECs with isolated MSH6 loss adds to a growing body of evidence that the detection of such tumours is particularly challenging using MSI. The fact that there is also a reduced signal in LS tumours suggests that MSI-based detection of MMRd in ECs from LS patients with MSH6 lesions is particularly difficult. Because of the relatively low number of LS and MSH6 tumours in both EC cohorts, we also analysed CRC cohorts and identified small but significant reductions in VAFs in both MSH6 and LS tumours.

An attenuated MSH6 MSI signal was first reported in CRC over 20 years ago [55] and is assumed to be due to functional redundancy between MSH6 and MSH3 [56,57]. The inclusion in early MSI panels of dinucleotide repeats, which MSH6 plays no role in repairing, may have accentuated this difference, but it has now been reported using a variety of MSI assays (including those using only mononucleotide repeats) and in a variety of disease contexts [16,32,58,59,60,61,62]. This is particularly noticeable in EC due to lower levels of MSI relative to CRC [30,31].

The evidence for a weaker MSI signal in LS-derived tumours is less well documented. An early comparison of inherited and sporadic CRC [63] reported a reduction in the number of unstable markers in the former (72% vs 87%), but as six out of the 10 MSI markers used were dinucleotide repeats, this may have been related to higher rates of MSH6 deficiency among the inherited CRCs. Why LS tumours may have a reduced MSI signal is unknown but could relate to differing tumorigenesis pathways and selection pressures. For example, MMRd tumours are highly immunogenic due to mutations in coding microsatellites leading to novel frameshift peptides [64,65], and increased T cell counts have been reported in the stroma, tumour, and invasive margins of MMRd/MSI-H LS derived CRCs [66,67], adenomas [68], and ECs [69,70], relative to sporadic tumours. Furthermore, the normal colonic mucosa of MMR mutation carriers develop numerous MMRd crypt foci not present in the general population [71], which could lead to immune surveillance, and LS cancers are often diagnosed before the sixth decade of life, the age at which the immune system begins to noticeably weaken [72]. Both could increase selection against increased frameshift peptide burden caused by high levels of MSI and result in the observed lower MSI marker VAFs within LS tumours.

## 5. Conclusions

Our re-analysis of clinical trial cohorts suggests that the Newcastle_MSI assay is more sensitive than Promega MSI V1.2 but further supports the prioritisation of IHC for LS detection in ECs due to their reduced MSI signal relative to CRC, which is particularly pronounced within inherited and sporadic *MSH6*-mutated tumours. The results also highlight how differences in IHC deployment, as well as tumour cell enrichment prior to molecular analyses, can impact assay concordance. However, the use of IHC in isolation is also known to result in missed LS cases regardless of pathology expertise [19,73,74] and will result in some cases suitable for immune checkpoint inhibitor treatment being missed. A dual approach combining upfront IHC and MSI testing could provide optimal outcomes, particularly in healthcare systems like the UK’s where low-cost genomic testing operates alongside a histopathology service with documented staffing challenges [75,76].

## Figures and Tables

**Table 1:** Overview of original IHC and MSI assay results in cohorts analysed. Overall concordance between assays is shown (Man = Manchester).

	% Ohio (n/Total)		% Man (n/Total)	
MMRd	49%	(97/196)	56%	(93/165)
MSI-H	48%	(95/196)	39%	(64/165)
Concordance	96%	(188/196)	78%	(129/165)

**Table 2:** Summary of original and 24-marker CRC-trained NCL_MSI assay results. Concordance between all assays is shown, together with the sensitivity and specificity of both MSI assays relative to IHC in training (Train), validation (Val), and merged datasets. Numbers of MMRd/MMRp samples are shown in parenthesis.

Cohort		Ohio		Manchester		
Dataset	Train	Val	Merged	Train	Val	Merged
IHC Result	(45/51)	(52/48)	(97/99)	(42/38)	(51/34)	(93/72)
A. Original Analyses						
Concordance MSI v IHC	95%	97%	96%	73%	82%	78%
Sensitivity MSI v IHC	96%	94%	95%	57%	71%	65%
Specificity MSI v IHC	94%	100%	97%	89%	100%	94%
B. NCL 24 Marker Panel						
Concordance NCL_MSI v MSI	97%	98%	97%	83%	89%	86%
Concordance NCL_MSI v IHC	94%	95%	94%	85%	84%	84%
Sensitivity NCL_MSI v IHC	93%	92%	93%	76%	76%	76%
Specificity NCL_MSI v IHC	94%	98%	96%	95%	94%	94%

**Table 3:** Tumours with known pathogenic germline MMR gene mutations. Results of IHC and MSI assays are shown. Cohorts—O/M = Ohio/Manchester, T/V = Training/Validation; Indicates Germline Testing = Assay identified MMRd/MSI in tumour.

Sample	Cohort	IHC Loss	Promega MSI	NCL_MSI Score	NCL_MSI Status	Indicates Germline Testing IHC Promega NCL		
ECT176	OT	MSH6	MSI-H	13.1	MSI-H	Y	Y	Y
ECT184	OT	MSH2/MSH6	MSI-H	15.7	MSI-H	Y	Y	Y
ECV69	OV	MSH6	MSI-H	17.7	MSI-H	Y	Y	Y
ECV100	OV	MSH2/MSH6	MSI-H	16.9	MSI-H	Y	Y	Y
ECT192	OV	No Loss	MSI-H	39.2	MSI-H	N	Y	Y
ECT102	OV	MSH6	MSS	−1.1	MSS	Y	N	N
PET256	MV	MLH1/PMS2	MSI-H	10.2	MSI-H	Y	Y	Y
PET16	MT	MLH1/PMS2	MSI-H	37.2	MSI-H	Y	Y	Y
PET61	MV	MSH6/MSH2	MSI-H	45.7	MSI-H	Y	Y	Y
PET215	MV	MSH6	MSI-H	26.2	MSI-H	Y	Y	Y
PET173	MV	PMS2	MSI-H	28.2	MSI-H	Y	Y	Y
PET213	MV	MSH6	MSI-H	30.3	MSI-H	Y	Y	Y
PET255	MT	MSH6	MSS	11.4	MSI-H	Y	N	Y
PET31	MT	MSH6	MSS	−19.1	MSS	Y	N	N
PET128	MT	MSH6	MSI-L	−24.6	MSS	Y	N	N
165BRC	MT	MSH6/MSH2	MSS	53.0	MSI-H	Y	N	Y
882BRC	MT	PMS2	MSS	−15.6	MSS	Y	N	N
PET241	MV	MSH6	MSI-H	−14.8	MSS	Y	Y	N
PRE011	MV	MSH6	MSS	−21.0	MSS	Y	N	N
PET96	MV	MSH6	MSS	−20.0	MSS	Y	N	N

## Data Availability

All FASTQ files are available from the EMBL-EBI European Nucleotide Archive, Project Accession reference PRJEB79220.

## Supplementary Materials

The following supporting information can be downloaded at: https://www.mdpi.com/article/10.3390/cancers16233970/s1: Table S1: Summary of all tumour cohorts analysed; Table S2: Details of markers, marker sets and samples analysed; Table S3: MIP sequence of additional MSI markers used to analyse CRCs. Table S4: Sample numbers received, processed and analysed by cohort; Table S5: Impact of classifier retraining on assay concordance and sensitivity/specificity with respect to IHC; Table S6. Details of Ohio cohort with original assay results, NCL_MSI scores and concordance patterns; Table S7: Details of Manchester cohort with original assay results, NCL_MSI scores and concordance patterns; Table S8: *MLH1* Promoter hypermethylation and NCL_MSI (24-marker, CRC-trained) classification relative to concordance with original Manchester assay results.
